# Results of a Global Survey of Experts to Categorize the Suitability of Interventions for Inclusion in School Health Services

**DOI:** 10.1016/j.jadohealth.2021.05.008

**Published:** 2021-12

**Authors:** Mary Louisa Plummer, Ace Chan, Kid Kohl, Ashley B. Taylor, Valentina Baltag, Elizabeth Saewyc, David Anthony Ross

**Affiliations:** a Department of Maternal, Newborn, Child and Adolescent Health and Ageing, World Health Organization (WHO), Geneva 27, Switzerland; b Stigma and Resilience Among Vulnerable Youth Centre (SARAVYC), University of British Columbia (UBC), Vancouver Campus, Vancouver, British Columbia, Canada; c SARAVYC and School of Nursing, UBC, Vancouver Campus, Vancouver, British Columbia, Canada

**Keywords:** Adolescent health, Child health, Global survey, Health-promoting schools, Online survey, School health, School health services, World Health Organization

## Abstract

**Purpose:**

This global survey of experts assessed the suitability of different health-related interventions for inclusion in school health services (SHSs) to inform development of the World Health Organization global guideline on SHSs.

**Methods:**

A review of 138 global World Health Organization publications identified 406 health service interventions for 5- to 19-year-old individuals. These were consolidated, pretested, and pilot-tested in a questionnaire as 86 promotion, prevention, care, or treatment interventions. A total of 1,293 experts were identified through purposive sampling of journal databases and professional networks. In July 2019, experts were invited to complete the questionnaire online in Arabic, Chinese, English, French, Russian, or Spanish. Respondents categorized each intervention as essential, highly suitable, suitable, or unsuitable in SHSs (everywhere or in certain geographic areas only). They could also suggest interventions.

**Results:**

Interventions categorized most often as “Essential in SHSs everywhere” (70%–80%) are related to health promotion and health education. Clinical interventions categorized most often in this way (60%–68%) are related to immunization, screening, assessment, and general care. Interventions categorized most often as “Essential in SHSs in certain geographic areas only” (27%–49%) are related to immunization, mass drug administration, and health promotion. Interventions categorized most often as “Unsuitable in SHSs anywhere” (12%–14%) are related to screening of noncommunicable conditions. There were no important regional differences. Of 439 respondents from 81 countries, 188 suggested 378 additional interventions. Question order effect and/or purposive sampling biases may have influenced both quantitative and qualitative results for different types of intervention.

**Conclusions:**

Favorable responses to almost all interventions supported their World Health Organization guideline inclusion but provided little guidance for intervention prioritization.

**Implications and Contribution**

Few studies examine school health services (SHSs) globally. This World Health Organization survey of 439 experts from 81 countries assessed the suitability of 86 interventions for inclusion in SHSs involving a health worker. Favorable responses to almost all interventions supported their possible use in SHSs but provided little guidance for intervention prioritization.

School-aged children and adolescents have significant health service needs but often do not access needed or desired health services [[1], [2], [3]]. Given high rates of school enrollment and estimates that students average 7,590 hours in the classroom during primary and lower secondary school, schools are the only platform that reaches most children and adolescents on a regular basis globally [[4], [5], [6]]. Most countries have some form of school health services (SHSs), but such programs may not be evidence-based and/or may not be implemented in an efficient way [4]. In addition, current SHSs are often limited by historical traditions and incremental development rather than informed choice reflecting changing priorities [7,8]. While numerous studies have focused on the quality and content of SHSs in specific countries – particularly in upper-middle-income and high-income countries – relatively few have assessed SHSs globally and/or focused on SHSs in low- and lower-middle-income countries [9].

This article describes the methodology and results of a global survey of expert opinion on the relative suitability of interventions for inclusion within SHSs. The survey focused on SHSs that are provided to students enrolled in primary or secondary education by a health worker, either within school premises (school-based services) or in specially designated facilities outside of schools (e.g., community health centers) that serve multiple schools (school-linked services). A health worker was defined as a person whose main function is to deliver health promotion, prevention, care, and treatment services, for example, a nurse or clinical psychologist, but not a teacher.

The survey was conducted to inform the development of the first World Health Organization (WHO) global guideline on SHSs [10]. The purpose of the guideline is to provide national governments and other stakeholders with guidance on the effectiveness, acceptability, and content of comprehensive SHSs involving a health worker. This effort is part of the WHO's broader “Make Every School a Health-Promoting School” initiative [[11], [12], [13], [14], [15], [16]]. The initiative outlines standards related to (1) government policies and resources; (2) a whole-school approach; (3) school governance and leadership; (4) the school community, including students and local communities; (5) a school curriculum supporting physical, social-emotional, and psychological health and well-being; (6) the school social-emotional environment; (7) the school physical environment; and (8) comprehensive school-based or school-linked health services [16]. For most of these eight standards, there is clear evidence-based guidance available that advises countries on how to address the standard in their school systems [[16], [17], [18], [19]]. To date, however, there has been no global guidance on SHSs that is supported by a rigorous review of the literature and of implementation experience.

## Methods

The rationale for the global online survey of experts was to assess the suitability of interventions for inclusion in SHSs to inform development of the WHO SHSs guideline. Importantly, this was not an academic research project but rather a practical, applied study with objectives to seek and consider the opinions of a large, diverse global sample of SHS experts in an efficient and affordable way.

The survey protocol, questionnaire, and expert list were developed by the WHO SHSs guideline secretariat and finalized based on input from the external Guideline Development Group (GDG). No formal ethics committee approval was required or sought by the WHO.

### Selection of experts to invite to participate in the survey

Email addresses of experts to invite to participate in the survey were compiled through purposive sampling [20,21]. The following methods were used:

1. A search of journal article databases using the term “school health” in April 2019, to identify one corresponding author for each article (n = 461). Specifically,
  a) PubMed articles from 2016 through April 2019 (n = 307).
  b) For each regional Index Medicus database, a saturation approach was used to identify 20 or more invitees. Databases were searched from the most recent year of publication available and backward by year, for up to three years. Databases, publication years searched, and final numbers of invitees were African region (2016–2018 January 2016 to December 2018, n = 12), Eastern Mediterranean region (January 2018 to April 2019; n = 30), region of the Americas/Pan American Health Organization (January 2018 to April 2019; n = 26), Southeast Asia region (January 2014 to December 2016; n = 28), and Western Pacific region (January 2018 to April 2019; n = 23). The search of the African Index Medicus did not result in enough possible invitees, so a supplemental search of Africa Journals OnLine was conducted (January 2018 to April 2019; n = 35).
2. Inclusion of the WHO SHSs guideline expert network, that is, GDG members and all other candidates nominated for the GDG, WHO Steering Group members, and selected headquarter and regional staff of WHO and the United Nations Educational, Scientific and Cultural Organization (UNESCO) (n = 105).
3. Inclusion of experts proposed by members of the GDG and the WHO Steering Group from their professional networks (n = 745).

For the working list, record was made of invitees' first and last names, email address, reason for inclusion (e.g., corresponding author; referred by a GDG member), and school health area of expertise (based on job title and/or specific article coauthorship). For the vast majority, record was also made of their institutional affiliation, including institutional country, country income status, and the WHO region. These data provided a rough sense of invitee characteristics as the list was compiled; they were not, however, verified, so they could not be used for more formal analyses. For the final list, email duplicates were removed, resulting in 1,293 expert invitees.

### Questionnaire and protocol development

The survey focused on a list of interventions that was developed in two stages. First, an initial review was conducted of health service recommendations for 5- to-19-year-old individuals in global WHO publications (e.g., guidelines, strategies, standards for quality of care, guidance documents). The search and review began with the most recent WHO publications on a topic and worked backward by publication year using a saturation approach, to include the most recently published WHO recommendations on a topic. It identified 406 procedures and activities within 138 publications, more than three-quarters (78%) of which were published between 2010 and 2019. The 406 procedures and activities were consolidated and refined by the WHO secretariat and the GDG as a working list of 86 interventions.

A first English draft of the questionnaire was developed by WHO in the program LimeSurvey (LimeSurvey, Hamburg, Germany) and was pretested with WHO staff (n = 3), after which the WHO made minor changes to improve clarity of language, logic, flow, length, and online administration. A second draft of the LimeSurvey questionnaire began with clinical intervention questions, continued with nonclinical intervention questions and questions about SHSs staffing needs, and finished with optional respondent sociodemographic questions. This was pilot tested with the GDG (n = 18). Afterward, GDG members reviewed the planned survey protocol, the draft questionnaire, and the results of the pilot test, and instructed the WHO in revisions that should be made before administration of the survey. Minor changes to the questionnaire included (1) refinement of language (e.g., emphasizing SHSs staff supporting rather than leading care roles); (2) addition of interventions (e.g., oral health promotion, infectious disease outbreak management); and (3) removal of questions about staffing needs, as this required knowledge of human resources and was likely to highly depend on context. Major changes to the survey after the pilot test included (1) limiting the survey to being invitation-only to maximize input from people with technical knowledge of SHSs; (2) randomizing the order of sections to reduce possible question order effect, but if this was not feasible, then moving nonclinical questions (e.g., health education, health promotion) before clinical questions (e.g., care and treatment); and (3) making the survey available in the six WHO languages, that is, Arabic, Chinese, English, French, Russian, and Spanish.

Given limited resources and time, it was not logistically feasible to make multiple versions of a randomized questionnaire available in each of six languages, so it was decided to translate but not randomize questions. To do so, it was necessary to reprogram and administer the final survey in Qualtrics (Qualtrics, Provo, Utah, USA). This was carried out by the Stigma and Resilience among Vulnerable Youth Centre at the University of British Columbia. Translations were completed by Qualtrics staff, and translation quality checks were conducted by GDG members or WHO staff. The validity and reliability of the survey and questionnaire were not assessed beyond the pretest and pilot test.

### Final questionnaire and survey administration

The final questionnaire included questions about the suitability of 86 interventions for inclusion within SHSs. These were organized by 16 types of intervention with the following sequence: health promotion, health education, and other aspects of a health-promoting school, assessment, screening, mass drug administration and immunization, health counseling to promote well-being, health counseling to prevent problems, sexual and reproductive health preventive care, general care, and care specific to communicable diseases, noncommunicable conditions, injury and violence, sexual and reproductive health, mental health, and substance use. Respondents were required to answer questions on whether each of the 86 interventions was essential, highly suitable, suitable, or unsuitable for inclusion within SHSs and whether the selected categorization was applicable in SHSs everywhere or in certain geographic areas only. If respondents did not know how to answer a question, they could select “Do Not Know.”

Survey respondents also had the option to write in up to three additional interventions which they believed were essential to include in SHSs, specifying whether they thought they were essential in SHSs everywhere or in certain geographic areas only. Finally, respondents were asked to answer seven optional sociodemographic questions, three of which allowed one answer only (i.e., gender, age, education level) and four of which were the only multiple-choice questions in the questionnaire (i.e., nationality, geographic area of expertise, health area of expertise, and profession).

In July 2019, each expert was sent an email invitation from the lead WHO researcher with a brief explanation of the survey, a unique link to the online questionnaire, and a statement that the survey was being administered by the University of British Columbia and would be open for two weeks. Two email reminders were sent to all invitees during the survey. On opening the link, the Introduction and Instructions pages explained the structure of the questionnaire, the estimated time needed to complete it (45 minutes), and a statement that all responses would be kept confidential. The Definitions page clarified key terms, such as “health-promoting school” and “screening.” At the end of each of these pages, respondents needed to indicate they had read the page before moving to the next one. Survey participation was considered to indicate implicit consent.

### Data analysis

Completion of at least the first page of five questions was a prerequisite for inclusion of a questionnaire within the data set. Basic frequencies and cross-tabulation of data were calculated in Microsoft Excel and SPSS Statistics software. Data were not anonymized, but personal information was kept confidential. Data analysis encompassed all intervention categories but primarily focused on interventions that were most frequently categorized by respondents as “Essential in SHSs everywhere,” “Essential in SHSs in certain geographic areas only,” or “Unsuitable in SHSs anywhere,” to inform prioritization of interventions within the guideline. Separate analyses were performed for all interventions (n = 86) and for clinical interventions only (n = 66). Clinical interventions were defined and postcoded as (1) needing to be delivered by or supervised by a health worker (n = 59) or (2) possibly being delivered by a health worker or delegated to a teacher with health worker support or supervision (n = 7).

Written responses to open-ended questions about additional interventions were first translated into English by GDG members or WHO staff, if needed. New suggestions were organized under the 16 types of intervention, with subgroups created to reflect emergent themes.

## Results

In total, 439 survey invitees completed at least the first page of five questions, so their responses were included in the final data set, resulting in a response rate of 34%. A total of 417 (95%) respondents completed all 86 questions, almost all of whom also completed the optional sociodemographic questions. Two-thirds (65%) of respondents were women and 35% men. Respondents varied in age from 25 to 81 years. The mean age and median age were both 51 years.

Respondents from 81 countries completed the questionnaire. More than half (221 of 423 [52%]) of all respondents represented 12 countries: Philippines (56), United States (42), China (38), United Kingdom (26), Australia (18), Canada (13), India (13), South Africa (13), Tunisia (13), Germany (10), Jordan (9), and Sweden (9). The reported level of education was 63% medical doctor or doctorate of philosophy, 27% master's degree, 9% bachelor's degree, 1% associate's degree, and <1% secondary school completion. Table 1 summarizes survey respondent profession, region of nationality, region of expertise, and health area of expertise.

**Table 1:** Results of the global survey of expert opinion on school health services: Self-reported sociodemographic characteristics of respondents^a^

Sociodemographic characteristics of survey respondents (n = 439)			
Profession (N = 416; 729 responses)	Health area of expertise (N = 412; 1140 responses)	Region of nationality^b^ (N = 405; 412 responses)	Regional expertise (N = 404; 537 responses)
• 30% researcher/academic • 27% health practitioner • 15% program manager • 14% teacher or other educational professional • 8% policy maker • 5% other	• 28% child and adolescent health and development • 16% nutrition and/or physical activity • 14% noncommunicable diseases • 13% mental health (including self-harm) and/or substance use • 13% sexual and reproductive health • 11% communicable diseases • 6% unintentional injury and violence	• 29% Western Pacific • 23% European • 18% Americas • 14% African • 12% Eastern Mediterranean • 4% Southeast Asia	• 23% Southeast Asia • 22% African • 19% European • 14% Americas • 11% Western Pacific • 11% Eastern Mediterranean

a These four sociodemographic questions were optional and also were the only survey questions that allowed multiple-choice answers. As individual respondents could select more than one answer option, the number of responses for each question was greater than the number of respondents. All answers were included in analysis unless otherwise noted.

b Reported nationality was post-coded to identify the WHO region of nationality. Seven individuals had multiple regions of nationality and were randomly assigned to one of their regions only.

### Intervention categorization

Appendix 1 summarizes responses for each of the 86 intervention questions.

Almost half (41 of 86) of the interventions were identified as “Essential in SHSs everywhere” by most respondents. Overall, the interventions that were categorized most often as essential in SHSs everywhere related to health promotion and health education, as shown in Table 2. The top interventions were promotion of personal hygiene and handwashing with soap (80%), provision of sexual and reproductive health education (75%), promotion of health literacy (73%), health education about nutrition (73%), and promotion of oral health care (70%).

**Table 2:** Results of the global survey of expert opinion on school health services: The 20 interventions that respondents most frequently categorized as “Essential everywhere” for inclusion within school health services

No.	Survey question number and intervention	Total respondents N	Respondents N (%)	Type of intervention
1.	Q03. Promotion of personal hygiene and handwashing with soap	439	351 (80.0)	Health promotion
2.	Q14. Provision of sexual and reproductive health education	438	327 (74.7)	Health education
3.	Q02. Promotion of health literacy	439	322 (73.3)	Health promotion
4.	Q12. Provision of health education about nutrition	438	320 (73.1)	Health education
5.	Q04. Promotion of oral health care (e.g., daily toothbrushing; fluoride application to teeth; care seeking for pain relief)	439	309 (70.4)	Health promotion
6.	Q06. Promotion of increased physical activity and limited sedentary behavior	439	309 (70.4)	Health promotion
7.	Q05. Promotion of reduced consumption of sugar and sugar-sweetened beverages	439	305 (69.5)	Health promotion
8.	Q37. Administration of immunizations recommended for all children (e.g., diphtheria-tetanus-pertussis, hepatitis B, human papillomavirus [females only], measles, rubella)	429	292 (68.1)	Immunization and mass drug administration
9.	Q13. Provision of health education about physical activity	438	297 (67.8)	Health education
10.	Q17. Support for school policies on risk reduction and disease/injury prevention (e.g., prevention of adolescent pregnancy, bullying, school violence, and substance use)	436	294 (67.4)	Other aspects of a health-promoting school
11.	Q24. Screening for vision problems	434	292 (67.3)	Screening
12.	Q16. Support for school policies on health promotion (e.g., related to chronic conditions, hygiene, mental health, and nutrition)	436	291 (66.7)	Other aspects of a health-promoting school
13.	Q15. Support for a health-promoting curriculum (e.g., curriculum-based sexuality education; curriculum on nutrition and physical activity)	438	292 (66.7)	Health education
14.	Q56. Provision of first aid, that is, identification and prioritization of problems, provision of immediate care, and referral for full medical treatment, if required (e.g., acute conditions such as asthma, diabetes, and seizures; bleeding or injury; mental health concerns, including self-harm; life-threatening allergy; poisoning and envenoming; and substance abuse)	422	278 (65.9)	General care
15.	Q01. Promotion of timely care seeking from an appropriate provider	439	288 (65.6)	Health promotion
16.	Q09. Promotion of menstrual hygiene management	439	288 (65.6)	Health promotion
17.	Q08. Promotion of adequate sleep	439	287 (65.4)	Health promotion
18	Q72. Referral and support for victims of violence (e.g., child abuse and neglect by parents or other caregivers; collective violence; gender-based or sexual violence; harmful cultural practices; violence among adolescents; and violence by intimate partners)	418	269 (64.4)	Injury and violence
19.	Q25. Screening for hearing problems	434	279 (64.3)	Screening
20	Q81. Referral and support for management of suicide risk/self-harm	418	259 (62.0)	Mental health care

Clinical interventions that were categorized most often as essential in SHSs everywhere related to immunization, screening, assessment, and general care. The top five specific interventions were administration of immunizations recommended for all children (68%), screening for vision problems (67%), provision of first aid (66%), screening for hearing problems (64%), and identification of developmental difficulties and disabilities (60%).

Interventions that were most often categorized as only essential in SHSs in certain geographic areas primarily related to immunization, mass drug administration and health promotion, followed by screening and referral for communicable diseases and noncommunicable conditions. The top five interventions in this category were administration of immunizations recommended for children residing in certain areas (49%), provision and promotion of use of insecticide-treated bed nets (48%), administration of immunizations recommended for children in some high-risk populations (44%), mass drug administration (38%), and promotion of use of sunscreen to prevent sunburn and skin cancer (27%).

None of the 86 interventions were categorized as being unsuitable for inclusion in SHSs anywhere by more than a small proportion of the respondents (0%–14%). For 81% of the interventions, only 0%–4% of respondents selected the option “Unsuitable in SHSs anywhere.” The interventions most commonly categorized as “Unsuitable in SHSs anywhere” were mainly related to noncommunicable disease screening, although specific interventions from other healthcare categories (i.e., general care, sexual and reproductive health preventive care, mental health, injury, and immunization) were also placed in this category by some respondents. The top interventions in this category were screening for hypertension (14%), administration of over-the-counter and prescribed medications (13%), and screening for diabetes (12%), scoliosis (12%), and other chronic conditions that may be undiagnosed (12%). Notably, 2 to 3 times the proportion of respondents categorized each of these interventions as “Essential in SHSs everywhere” or “Essential in SHSs in certain geographic areas only.” For example, while 12% of respondents categorized “Screening for diabetes” as “Unsuitable in SHSs anywhere,” 34% of respondents categorized it as “Essential in SHSs” (26% “everywhere”; 8% “in certain geographic areas only”).

### Intervention categorization by the WHO region and profession

**Figure 1:**
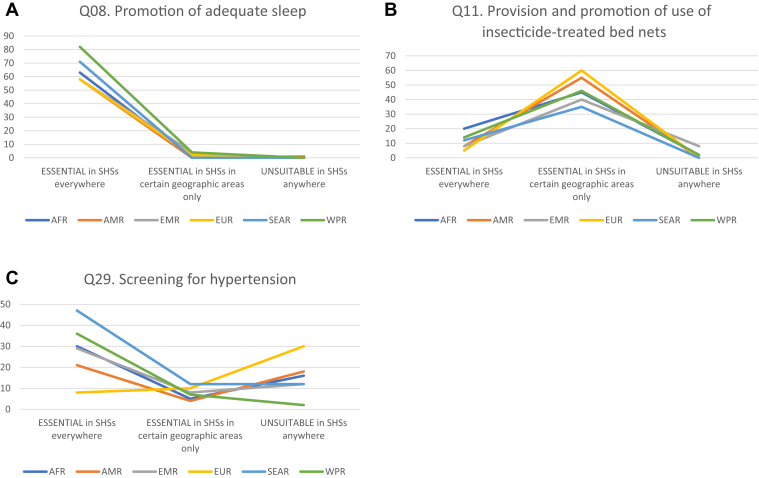
Results of the global survey of expert opinion on school health services: Examples of three common patterns in survey responses, by region: (A) High “Essential in SHSs everywhere”; (B) High “Essential in SHSs in certain geographic areas only”; and (C) High “Unsuitable in SHSs anywhere”^a^. Key: AFR = African region; AMR = Region of the Americas; EMRO = Eastern Mediterranean region; EUR = European region; SEAR = Southeast Asia region; SHSs = school health services; WPR = Western Pacific Region. ^a^ Responses for “Highly suitable everywhere,” “Highly suitable in certain geographic areas only,” “Suitable everywhere,” “Suitable in certain geographic areas only,” and “Do not know” can be seen in Appendix 1.

The interventions that were most often categorized as (1) “Essential in SHSs everywhere,” (2) “Essential in SHSs in certain geographic areas only,” or (3) “Unsuitable in SHSs anywhere” were very similar across regions, although the proportions of respondents determining these categorizations varied by the region. Figure 1 shows common patterns of response for interventions categorized in these ways, illustrated by (1) promotion of adequate sleep, (2) provision and promotion of insecticide-treated bed nets, and (3) screening for hypertension (Appendix 1, Q08, Q11, and Q29, respectively).

Subgroup analysis by profession found categorization of interventions was very similar by profession across all interventions.

### Suggestions of additional essential interventions

A total of 188 (43%) respondents wrote 378 intervention suggestions, 63% of which were in English, 22% in French, 7% in Spanish, 6% in Chinese, 2% in Arabic, and none in Russian. The vast majority of these interventions were proposed for inclusion within SHSs everywhere. Approximately half was related to health promotion, health education, and other aspects of a health-promoting school, with most in the latter category. Most of the remaining suggestions were related to assessment, general care, noncommunicable condition care, and mental health care. Table 3 lists the interventions that four or more respondents suggested. Appendix 2 provides examples of the 119 interventions that were suggested by 1–3 respondents. Within the general care category, an overarching theme was provision of routine, basic services, such as management of student medical equipment (e.g., tracheotomy, ventilator, G-tube, blood glucose monitor), fever treatment, and administration of rescue medications and procedures (e.g., EpiPen injections and albuterol inhalers for patients with anaphylaxis or asthma attacks).

**Table 3:** Results of the global survey of expert opinion on school health services: Additional interventions suggested as essential in school health services by four or more respondents, by type of intervention^a^

Health promotion
1. Promotion of safe and/or restricted use of mobile phones and the internet
2. Address bullying and peer harassment
3. Promotion of disability acceptance/support and integration
Health education
4. Education on the safe use of technology/Internet, for example, social networks, video games, pornography, and addiction to mobile phones, selfies, and/or the Internet^b^
5. Life skills education^c^ and/or a formal social and emotional learning curriculum
6. Sex education as “highly essential”^c^
7. Education about road safety^c^
8. Education about parenting skills and responsible parenthood
9. Education about gender equality^c^
10. Education about climate change, population growth, the environment/ecology, and health, and what can be done at the individual level, for example, school garden
Other aspects of a health-promoting school
11. Collaboration with teachers and other school staff in multidisciplinary health services and classroom work (e.g., psychologist, social worker, special education, occupational therapy)^b^
12. Support for an adequate supply of clean water for drinking, cleaning and flushing, and adequate, safe sanitation^b^^,^^c^^,^^d^
13. Routine health inspection and regular walk-through in all areas of the school to assess risks and ensure a safe and healthful environment, conducive to learning and free from hazards, for example, electrical safety; installing sidewalks and pathway railings for injury prevention; sanitary canteen, classrooms, and residencies (boarding schools)
14. Universal and consistent provision of healthy and nutritionally adequate food and drink throughout education, especially for severely wasted students, including the importance of food production^c^
15. Ensure an accessible environment for all in the classroom or in physical education, for example, visual aids, desks, chairs^c^
16. Training of all school actors on school health care interventions, for example, training of teachers and other school staff (such as cooks) in first aid, basic health care (for schools without a health worker), general hygiene, dental hygiene, food hygiene, nutrition, and/or recognition and management of common mental health problems^c^
17. Integration of children with special needs (e.g., epilepsy, neurodevelopmental disorders) in school, and specialized services to address their learning, behavioral, and other needs
18. Ensure students have healthy recreational and/or outdoor activities, for example, sports development programs for athletes; playgrounds; yoga or meditation activity 10–15 minutes/day; and assessment of the quality of physical education/sports provided by the school
19. On-going engagement with parents, families, and local community leaders, e.g., political, traditional, religious leaders
Assessment
20. Phonoaudiological evaluation (e.g., hypotonic tongue, peribuccal muscles, atypical swallowing, mouth breathing, pronunciation problems, speech), for example, stuttering
Screening
21. Early screening of physical, behavioral, developmental and learning needs, for example, readiness on school entry, to allow for early intervention such as expressive and receptive speech language assessment at 4–5 years, or services related to autism spectrum disorder^b^
Health counseling to prevent problems
22. Address stress related to school and grades for adolescents,^c^ especially in low- and middle-income countries, for example, yoga, music therapy, or group sessions for moderating psychological stress through games
General care
23. Provide a referral system, especially to respond to cases from remote/difficult-to-access school communities^c^
Noncommunicable conditions care
24. Occupational therapy at all levels of the support continuum, for example, modification of activities and adaptation of the school environment to make it accessible for students with disabilities^b^^,^^c^
25. Specific nutrition interventions,^b^^,^^c^ for example, referral, support, and management for overweight and obesity; management and referral of moderate and severe acute malnutrition
26. Support and treatment services for children with chronic neurological conditions, for example, cerebral palsy, traumatic brain injury, epilepsy
27. Prevention, referral, and intervention for myopia^c^
28. Primary oral health care, for example, prevention and control of dental caries by topical fluorination or pit and groove sealing; fillings using atraumatic restorative treatment; nonsurgical extraction^b^^,^^d^
Sexual and reproductive health care
29. Contraceptive counseling and provision, if legally allowed in a country
Mental health care
30. Cognitive and environmental interventions for children with attention-deficit hyperactivity disorder, autism spectrum disorder, or developmental coordination disorder^c^

a All interventions were suggested for inclusion in school health services everywhere, unless otherwise noted.

b Suggested by six or more respondents.

c Suggested both for school health services everywhere and for those in certain geographic areas only.

d Suggested for school health services in certain geographic areas only.

## Discussion

This global survey of expert opinion on interventions for potential inclusion in SHSs succeeded in collecting data from a large and diverse sample, in terms of education, profession, health specialization, and geographic specialization (Table 1). The survey results seem plausible; for example, it is logical that the interventions most often categorized as “Essential in SHSs in certain geographic areas only” are those which target conditions that have a limited geographic range, owing to disease prevalence, population, resources, ecology, and/or climate (Appendix 1).

The survey results were useful to WHO SHSs guideline development in multiple ways. The high overall survey categorization of health promotion and health education interventions as “Essential in SHSs everywhere” indicates that these are important roles for health workers to perform within a school setting. This is consistent with the current scope of practice of health workers (e.g., licensed school nurses) and/or team-based SHSs in some countries, which explicitly includes these types of interventions [4]. The survey results further indicate that SHSs should be comprehensive and involve delivery of a wide range of services to students.

Importantly, despite the sizeable and diverse sample of respondents, all of the interventions in the questionnaire were categorized by a large majority of respondents as essential or suitable in SHSs. These findings are promising in that none of the interventions listed in the expert questionnaire received a strong negative response, so all could potentially be considered for inclusion within the SHSs guideline. This overwhelmingly positive response is not surprising, as the 86 interventions were based on general health service interventions for 5- to 19-year-old individuals published in global WHO sources. Nonetheless, validation by experts in all global regions is useful, particularly for issues that can be controversial. It is noteworthy, for example, that of 86 interventions, “Provision of sexual and reproductive health education” had the second highest categorization as “Essential in SHSs everywhere” (327 of 438 [75%]) (Table 2). Indeed, only 3 of 438 (1%) respondents identified this intervention as “Unsuitable in SHSs anywhere,” so it was not in the top 20 interventions in that category (Appendix 1).

However, these survey results also posed a challenge, as they did not provide much guidance for prioritization of interventions within the *WHO* guideline on SHSs [10]. In many contexts, especially in low- and middle-income countries, prioritization is critical as it may not be feasible to include dozens of different health interventions within SHSs. For example, in some settings, it may be more cost-effective to have nonclinical staff (e.g., teachers) carry out nonclinical interventions such as health education and health promotion. The overwhelmingly favorable response to most interventions in the survey was further complicated by the high response rate for write-in suggestions of essential interventions. After review, several of the new interventions were added to the pool included within the guideline, such as support for school policies around prevention and response to anaphylaxis; referral and support for overweight and obesity; and appropriate use of data at the population level for SHSs planning.

### Limitations of the research

The broad scope of this research surveying experts from all regions of the world, on all possible interventions that could be included within SHSs, meant that it was not possible to evaluate some important SHSs questions with subtlety and depth. For instance, the survey only marginally explored SHSs concerns specific to low-, middle-, and high-income settings, and it did not compare SHSs within governments with different healthcare systems. Similarly, the survey did not consider how SHSs needs differ at the primary and secondary school levels or how they may vary in settings with other sources of health education and health promotion (e.g., within schools or by nonprofit agencies). In addition, the 1,293 survey invitees were identified as “experts” through simple search criteria, and we were unable to otherwise evaluate respondents' capabilities to assess the suitability of a wide range of interventions at the global level or how their experience or sociodemographic biases may have influenced them.

There are several ways that question order biases and/or purposive sampling biases may have influenced the results of this survey. Question order effect may have contributed to the pattern of nonclinical interventions being the interventions most often categorized as “Essential in SHSs everywhere” [[22], [23], [24]]. Sixteen of the top 20 interventions categorized as “Essential in SHSs everywhere” do not need to be delivered by or supervised by a health worker, although they could be part of health worker practice, for example, support for school policies on risk reduction and disease/injury prevention. Five of the top seven interventions in this category were among the first six questions of the questionnaire. Similarly, the clinical interventions that were categorized most often as “Essential in SHSs everywhere” were among the first clinical interventions in the questionnaire. The possibility of response bias owing to question order (e.g., an initially affirmative response to all questions, followed by more subtle distinctions in later responses) could not be ruled out.

Purposive sampling biases related to the online databases searched, the term used, and the expert networks accessed may also have contributed to the relatively high “essential” categorization of nonclinical interventions [25,26]. Use of the broad term “school health” for the database search may have resulted in disproportionate inclusion of experts focused on the seven nonclinical standards of health-promoting schools (e.g., a school curriculum supporting student health) [16], as these standards may be far more represented than SHSs in school systems and the research literature.

Furthermore, the databases themselves may be inherently biased toward high-income country authors, as, historically, published research has focused on high-income countries disproportionately [27]. Our effort to address this potential bias by going beyond the PubMed database to search each of the regional Index Medicus databases was only partially successful, as even articles that focus on low- and middle-income countries often have a corresponding author from a high-income country [27]. Efforts to address possible high-income and/or North American and European country biases by relying on the professional networks of the WHO Steering Committee and GDG seem to have been somewhat effective but may have introduced other biases. For example, three GDG members who primarily work in the Philippines, China, and Tunisia accounted for more than one quarter of all survey invitees, likely contributing to those countries being among the most common respondent nationalities.

Finally, the main healthcare areas addressed in the write-in essential intervention suggestions (e.g., care for noncommunicable conditions) also were among the main health areas of expertise reported by respondents (Tables 1 and 3). If more respondents had had expertise in other areas, such as communicable diseases, injury, and violence, then related interventions may have been categorized as essential in SHSs more often. Importantly, such conditions disproportionately affect children and adolescents in low- and middle-income countries [1,3,28].

These survey limitations suggest that while the broad intervention categories and patterns are plausible and were useful in the general development of the *WHO* guideline on SHSs [10], caution should be taken when interpreting fine or subtle differences. If a similar survey were to be conducted in the future, biases may be reduced if the question order is randomized and the expert pool is selected for greater representativeness of geographic regions, country income status, healthcare areas of expertise, and other sociodemographic factors.

This global survey of experts was useful in validating interventions being considered for inclusion within the *WHO* guideline on SHSs [10]. The high overall categorization of health education and promotion interventions as “Essential in SHSs everywhere” indicates that these should be included in SHSs. Similarly, among clinical interventions, the high categorization of immunization, screening, assessment, and general care interventions as “Essential in SHSs everywhere” highlights their importance. However, the broad scope of the research and the possibility of the question order and/or sampling biases suggest that caution should be taken when interpreting fine or subtle differences in the survey results.

## Funding Sources

This work was supported by the Department for International Development, London, UK (Programme Grant 300350 to WHO: Improving Information and Accountability for Women's, Children's and Adolescents' Health).
