# Supplementary material for: Results of a Global Survey of Experts to Categorize the Suitability of Interventions for Inclusion in School Health Services
Source: J Adolesc Health. 2021 Dec;69(6):948–56. doi: 10.1016/j.jadohealth.2021.05.008 (PMC8631416; doi:10.1016/j.jadohealth.2021.05.008)
Supplement: Appendix 1 [file mmc1.pdf]

Results of a Global Survey of Experts to Categorize the Suitability of Interventions for Inclusion in School Health Services

Appendix 1

Categorization of interventions as essential, highly suitable, suitable, or unsuitable in school health services, either everywhere or in certain geographic areas only.

**TYPE OF INTERVENTION: Interventions in Questionnaire Order (Q01-Q86)**	**Total Respond-ents** N	**ESSENTIAL in SHSs**		**HIGHLY SUITABLE in SHSs**		**SUITABLE in SHSs**		**UNSUITABLE in SHSs anywhere** N (%)	**Do not know** N (%)
		**everywhere** N (%)	**in certain geographic areas only** N (%)	**everywhere** N (%)	**in certain geographic areas only** N (%)	**everywhere** N (%)	**in certain geographic areas only** N (%)		
**HEALTH PROMOTION**									
Q01. Promotion of timely care-seeking from an appropriate provider	439	288 (66)	27 (6)	68 (15)	11 (3)	24 (5)	12 (3)	4 (1)	5 (1)
Q02. Promotion of health literacy	439	322 (73)	22 (5)	63 (14)	6 (1)	23 (5)	0 (0)	2 (0)	1 (0)
Q03. Promotion of personal hygiene and handwashing with soap	439	351 (80)	21 (5)	39 (9)	7 (2)	18 (4)	2 (0)	1 (0)	1 (0)
Q04. Promotion of oral health care (e.g. daily tooth-brushing; fluoride	439	309 (70)	25 (6)	66 (15)	10 (2)	23 (5)	4 (1)	0 (0)	2 (0)

TYPE OF INTERVENTION: Interventions in Questionnaire Order (Q01-Q86)	Total Respond-ents N	ESSENTIAL in SHSs		HIGHLY SUITABLE in SHSs		SUITABLE in SHSs		UNSUITABLE in SHSs anywhere N (%)	Do not know N (%)
		everywhere N (%)	in certain geographic areas only N (%)	everywhere N (%)	in certain geographic areas only N (%)	everywhere N (%)	in certain geographic areas only N (%)		
application to teeth; care-seeking for pain relief)									
Q05. Promotion of reduced consumption of sugar and sugar-sweetened beverages	439	305 (69)	27 (6)	63 (14)	11 (3)	23 (5)	7 (2)	2 (0)	1 (0)
Q06. Promotion of increased physical activity and limited sedentary behaviour	439	309 (70)	35 (8)	56 (13)	15 (3)	19 (4)	4 (1)	0 (0)	1 (0)
Q07. Promotion of appropriate use of electronic screens	439	166 (38)	71 (16)	83 (19)	44 (10)	42 (10)	26 (6)	2 (0)	5 (1)
Q08. Promotion of adequate sleep	439	287 (65)	9 (2)	87 (20)	6 (1)	40 (9)	4 (1)	3 (1)	3 (1)
Q09. Promotion of menstrual hygiene management	439	288 (66)	33 (8)	68 (15)	18 (4)	21 (5)	5 (1)	4 (1)	2 (0)

TYPE OF INTERVENTION: Interventions in Questionnaire Order (Q01-Q86)	Total Respond-ents N	ESSENTIAL in SHSs		HIGHLY SUITABLE in SHSs		SUITABLE in SHSs		UNSUITABLE in SHSs anywhere N (%)	Do not know N (%)
		everywhere N (%)	in certain geographic areas only N (%)	everywhere N (%)	in certain geographic areas only N (%)	everywhere N (%)	in certain geographic areas only N (%)		
Q10. Promotion of use of sunscreen to prevent sunburn and skin cancer	439	112 (26)	117 (27)	57 (13)	78 (18)	23 (5)	43 (10)	8 (2)	1 (0)
Q11. Provision and promotion of use of insecticide-treated bed nets	439	47 (11)	211 (48)	19 (4)	96 (22)	5 (1)	47 (11)	8 (2)	6 (1)
**HEALTH EDUCATION**									
Q12. Provision of health education about nutrition	438	320 (73)	11 (3)	73 (17)	9 (2)	20 (5)	3 (1)	1 (0)	1 (0)
Q13. Provision of health education about physical activity	438	297 (68)	12 (3)	93 (21)	9 (2)	20 (5)	4 (1)	2 (0)	1 (0)
Q14. Provision of sexual and reproductive health education	438	327 (75)	17 (4)	60 (14)	7 (2)	17 (4)	5 (1)	3 (1)	2 (0)
Q15. Support for a health-promoting curriculum (e.g. curriculum-based	438	292 (67)	16 (4)	96 (22)	6 (1)	23 (5)	2 (0)	2 (0)	2 (0)

TYPE OF INTERVENTION: Interventions in Questionnaire Order (Q01-Q86)	Total Respond-ents N	ESSENTIAL in SHSs		HIGHLY SUITABLE in SHSs		SUITABLE in SHSs		UNSUITABLE in SHSs anywhere N (%)	Do not know N (%)
		everywhere N (%)	in certain geographic areas only N (%)	everywhere N (%)	in certain geographic areas only N (%)	everywhere N (%)	in certain geographic areas only N (%)		
sexuality education; curriculum on nutrition and physical activity)									
**OTHER ASPECTS OF A HEALTH-PROMOTING SCHOOL**									
Q16. Support for school policies on health promotion (e.g. related to chronic conditions, hygiene, mental health, and nutrition)	436	291 (67)	21 (5)	83 (19)	6 (1)	29 (7)	4 (1)	0 (0)	2 (0)
Q17. Support for school policies on risk reduction and disease/injury prevention (e.g. prevention of adolescent pregnancy, bullying, school violence, and substance use)	436	294 (67)	20 (5)	88 (20)	14 (3)	15 (3)	4 (1)	0 (0)	1 (0)

TYPE OF INTERVENTION: Interventions in Questionnaire Order (Q01-Q86)	Total Respond-ents N	ESSENTIAL in SHSs		HIGHLY SUITABLE in SHSs		SUITABLE in SHSs		UNSUITABLE in SHSs anywhere N (%)	Do not know N (%)
		everywhere N (%)	in certain geographic areas only N (%)	everywhere N (%)	in certain geographic areas only N (%)	everywhere N (%)	in certain geographic areas only N (%)		
Q18. Support for other aspects of a health-promoting school (e.g. nutritional content of school feeding programmes; inspection of food safety and the school environment)[a]	436	246 (56)	23 (5)	100 (23)	17 (4)	34 (8)	10 (2)	3 (1)	3 (1)
Q19. Collection, analysis, and use of data on school health service utilization and quality of care, to monitor performance and support quality improvement[a]	436	258 (59)	25 (6)	94 (22)	1 (0)	44 (10)	8 (2)	3 (1)	3 (1)
**ASSESSMENT**									
Q20. Assess and ensure compliance with school entry health requirements	435	248 (57)	21 (5)	78 (18)	21 (5)	40 (9)	10 (2)	11 (3)	6 (1)

TYPE OF INTERVENTION: Interventions in Questionnaire Order (Q01-Q86)	Total Respond-ents N	ESSENTIAL in SHSs		HIGHLY SUITABLE in SHSs		SUITABLE in SHSs		UNSUITABLE in SHSs anywhere N (%)	Do not know N (%)
		everywhere N (%)	in certain geographic areas only N (%)	everywhere N (%)	in certain geographic areas only N (%)	everywhere N (%)	in certain geographic areas only N (%)		
(e.g. medical history, comprehensive physical examination, and immunization)[a]									
Q21. Routine preventive health check-ups (e.g. to assess physical growth, motor development, social and emotional maturation, and feeding and sleep problems, and to offer appropriate care or referrals)[a]	435	221 (51)	27 (6)	97 (22)	18 (4)	45 (10)	21 (5)	4 (1)	2 (0)
Q22. Conduct HEADSSS assessments, i.e. assessments of risk behaviours related to Home, Education/Employment, Eating, Activity,	435	199 (46)	29 (7)	112 (26)	18 (4)	43 (10)	21 (5)	7 (2)	6 (1)

TYPE OF INTERVENTION: Interventions in Questionnaire Order (Q01-Q86)	Total Respond-ents N	ESSENTIAL in SHSs		HIGHLY SUITABLE in SHSs		SUITABLE in SHSs		UNSUITABLE in SHSs anywhere N (%)	Do not know N (%)
		everywhere N (%)	in certain geographic areas only N (%)	everywhere N (%)	in certain geographic areas only N (%)	everywhere N (%)	in certain geographic areas only N (%)		
Drugs, Sexuality, Safety, and Suicidal thinking / Depression (i.e. to detect adolescent health and development problems; if their behaviour puts them at risk of negative health outcomes; and important factors in their environment that increase the likelihood of these behaviours)[a]									
Q23. Identification of developmental difficulties and disabilities[a]	435	262 (60)	14 (3)	87 (20)	12 (3)	41 (9)	12 (3)	4 (1)	3 (1)
**SCREENING**									
Q24. Screening for vision problems[a]	434	292 (67)	18 (4)	83 (19)	6 (1)	27 (6)	4 (1)	4 (1)	0 (0)
Q25. Screening for hearing problems[a]	434	279 (64)	16 (4)	83 (19)	10 (2)	32 (7)	6 (1)	5 (1)	3 (1)

TYPE OF INTERVENTION: Interventions in Questionnaire Order (Q01-Q86)	Total Respond-ents N	ESSENTIAL in SHSs		HIGHLY SUITABLE in SHSs		SUITABLE in SHSs		UNSUITABLE in SHSs anywhere N (%)	Do not know N (%)
		everywhere N (%)	in certain geographic areas only N (%)	everywhere N (%)	in certain geographic areas only N (%)	everywhere N (%)	in certain geographic areas only N (%)		
Q26. Screening for oral health problems[a]	434	260 (60)	23 (5)	82 (19)	10 (2)	40 (9)	11 (3)	7 (2)	1 (0)
Q27. Screening for nutrition problems (e.g. anaemia, malnutrition, obesity)[a]	434	246 (57)	33 (8)	84 (19)	21 (5)	30 (7)	13 (3)	5 (1)	2 (0)
Q28. Screening for diabetes[a]	434	113 (26)	35 (8)	89 (21)	25 (6)	68 (16)	46 (11)	53 (12)	5 (1)
Q29. Screening for hypertension[a]	434	117 (27)	30 (7)	79 (18)	22 (5)	75 (17)	40 (9)	62 (14)	9 (2)
Q30. Screening for scoliosis[a]	434	127 (29)	26 (6)	84 (19)	17 (4)	85 (20)	39 (9)	50 (12)	6 (1)
Q31. Screening for other chronic conditions that may be undiagnosed[a]	434	90 (21)	32 (7)	98 (23)	18 (4)	84 (19)	44 (10)	54 (12)	14 (3)
Q32. Screening for infectious diseases (e.g. tuberculosis; neglected tropical diseases, such as Chagas disease)[a]	434	115 (26)	114 (26)	34 (8)	59 (14)	22 (5)	70 (16)	15 (3)	5 (1)

TYPE OF INTERVENTION: Interventions in Questionnaire Order (Q01-Q86)	Total Respond-ents N	ESSENTIAL in SHSs		HIGHLY SUITABLE in SHSs		SUITABLE in SHSs		UNSUITABLE in SHSs anywhere N (%)	Do not know N (%)
		everywhere N (%)	in certain geographic areas only N (%)	everywhere N (%)	in certain geographic areas only N (%)	everywhere N (%)	in certain geographic areas only N (%)		
Q33. Screening for mental health concerns (e.g. to identify students at risk of poor mental health outcomes and/or who may need monitoring or referral)[a]	432	244 (56)	19 (4)	91 (21)	8 (2)	34 (8)	19 (4)	13 (3)	4 (1)
Q34. Screening for maltreatment by a parent or guardian[a]	432	224 (52)	32 (7)	94 (22)	8 (2)	37 (9)	17 (4)	16 (4)	4 (1)
Q35. Screening for exposure to intimate partner violence[a]	432	176 (41)	39 (9)	98 (23)	17 (4)	50 (12)	20 (5)	25 (6)	7 (2)
Q36. Screening for substance use (e.g. tobacco, alcohol, illicit drugs)[a]	432	200 (46)	27 (6)	100 (23)	21 (5)	44 (10)	14 (3)	20 (5)	6 (1)
**IMMUNIZATION AND MASS DRUG ADMINISTRATION**									

TYPE OF INTERVENTION: Interventions in Questionnaire Order (Q01-Q86)	Total Respond-ents N	ESSENTIAL in SHSs		HIGHLY SUITABLE in SHSs		SUITABLE in SHSs		UNSUITABLE in SHSs anywhere N (%)	Do not know N (%)
		everywhere N (%)	in certain geographic areas only N (%)	everywhere N (%)	in certain geographic areas only N (%)	everywhere N (%)	in certain geographic areas only N (%)		
Q37. Administration of immunizations recommended for all children (e.g. diphtheria-tetanus-pertussis, hepatitis B, human papillomavirus (females only), measles, rubella)[a]	429	292 (68)	23 (5)	51 (12)	18 (4)	18 (4)	17 (4)	5 (1)	5 (1)
Q38. Administration of immunizations recommended for children residing in certain regions (e.g. Japanese encephalitis)[a]	429	53 (12)	210 (49)	17 (4)	77 (18)	9 (2)	43 (10)	9 (2)	11 (3)
Q39. Administration of immunizations recommended for children in some high-risk populations (e.g. cholera,	429	76 (18)	187 (44)	23 (5)	78 (18)	10 (2)	41 (10)	6 (1)	8 (2)

TYPE OF INTERVENTION: Interventions in Questionnaire Order (Q01-Q86)	Total Respond-ents N	ESSENTIAL in SHSs		HIGHLY SUITABLE in SHSs		SUITABLE in SHSs		UNSUITABLE in SHSs anywhere N (%)	Do not know N (%)
		everywhere N (%)	in certain geographic areas only N (%)	everywhere N (%)	in certain geographic areas only N (%)	everywhere N (%)	in certain geographic areas only N (%)		
dengue, hepatitis A, meningococcal, rabies, typhoid)[a]									
Q40. Administration of immunizations recommended for children with specific health conditions (e.g. seasonal influenza for children with specific conditions)[a]	429	116 (27)	85 (20)	76 (18)	47 (11)	32 (7)	38 (9)	26 (6)	9 (2)
Q41. Mass drug administration (e.g. for soil-transmitted helminths, schistosomiasis, trachoma, malaria, lymphatic filariasis)[a]	429	75 (17)	163 (38)	14 (3)	74 (17)	7 (2)	64 (15)	17 (4)	15 (3)
Q42. Iron, folic acid and other micronutrient supplementation[a]	429	120 (28)	89 (21)	55 (13)	54 (13)	23 (5)	59 (14)	17 (4)	12 (3)

TYPE OF INTERVENTION: Interventions in Questionnaire Order (Q01-Q86)	Total Respond-ents N	ESSENTIAL in SHSs		HIGHLY SUITABLE in SHSs		SUITABLE in SHSs		UNSUITABLE in SHSs anywhere N (%)	Do not know N (%)
		everywhere N (%)	in certain geographic areas only N (%)	everywhere N (%)	in certain geographic areas only N (%)	everywhere N (%)	in certain geographic areas only N (%)		
**HEALTH COUNSELLING TO PROMOTE WELL-BEING**									
Q43. Psychosocial intervention to promote well-being and functioning (e.g. encouraging and/or assisting a child to: get enough sleep; eat regularly; be physically active; participate in social activities; spend time with trusted friends and family; avoid the use of alcohol, drugs, and nicotine; and develop interpersonal, emotion regulation, problem-solving and stress management skills)[a]	426	252 (59)	12 (3)	96 (23)	10 (2)	38 (9)	12 (3)	5 (1)	1 (0)

TYPE OF INTERVENTION: Interventions in Questionnaire Order (Q01-Q86)	Total Respond-ents N	ESSENTIAL in SHSs		HIGHLY SUITABLE in SHSs		SUITABLE in SHSs		UNSUITABLE in SHSs anywhere N (%)	Do not know N (%)
		everywhere N (%)	in certain geographic areas only N (%)	everywhere N (%)	in certain geographic areas only N (%)	everywhere N (%)	in certain geographic areas only N (%)		
Q44. Counselling and care related to a child's physical and psychosocial development (e.g. puberty, skin changes, body image, hygiene)[b]	426	230 (54)	10 (2)	113 (27)	7 (2)	49 (12)	11 (3)	4 (1)	2 (0)
Q45. Counselling and support for a child's caregiver related to the child's physical and psychosocial development (e.g. nutrition, physical activity, puberty, positive development in adolescence, sexual and reproductive health)[a]	426	191 (45)	9 (2)	129 (30)	15 (4)	62 (15)	12 (3)	6 (1)	2 (0)
**HEALTH COUNSELLING TO PREVENT PROBLEMS**									

TYPE OF INTERVENTION: Interventions in Questionnaire Order (Q01-Q86)	Total Respond-ents N	ESSENTIAL in SHSs		HIGHLY SUITABLE in SHSs		SUITABLE in SHSs		UNSUITABLE in SHSs anywhere N (%)	Do not know N (%)
		everywhere N (%)	in certain geographic areas only N (%)	everywhere N (%)	in certain geographic areas only N (%)	everywhere N (%)	in certain geographic areas only N (%)		
Q46. Counselling on nutrition, physical activity, and a management plan[b]	423	197 (47)	17 (4)	107 (25)	13 (3)	62 (15)	17 (4)	6 (1)	4 (1)
Q47. Counselling on tobacco, alcohol, and other substance use[b]	423	203 (48)	18 (4)	104 (25)	18 (4)	55 (13)	15 (4)	7 (2)	3 (1)
Q48. Counselling to prevent common injuries (e.g. how to prevent unintentional injuries in the home, while playing or engaged in sports, and on roads)[b]	423	182 (43)	12 (3)	120 (28)	11 (3)	71 (17)	9 (2)	13 (3)	5 (1)
Q49. Counselling to prevent intimate partner violence (e.g. universal information provided on prevention of intimate partner violence; selected	423	163 (39)	28 (7)	120 (28)	17 (4)	57 (13)	24 (6)	9 (2)	5 (1)

TYPE OF INTERVENTION: Interventions in Questionnaire Order (Q01-Q86)	Total Respond-ents N	ESSENTIAL in SHSs		HIGHLY SUITABLE in SHSs		SUITABLE in SHSs		UNSUITABLE in SHSs anywhere N (%)	Do not know N (%)
		everywhere N (%)	in certain geographic areas only N (%)	everywhere N (%)	in certain geographic areas only N (%)	everywhere N (%)	in certain geographic areas only N (%)		
therapeutic approaches for high-risk youth)[b]									
Q50. Counselling to prevent other kinds of violence, including sexual violence, gender-based violence, and gang violence (e.g. universal information provided on prevention of violence and abuse; selected therapeutic approaches for high-risk youth)[b]	423	203 (48)	24 (6)	105 (25)	12 (3)	52 (12)	17 (4)	4 (1)	6 (1)
**SEXUAL AND REPRODUCTIVE HEALTH PREVENTIVE CARE**									
Q51. Contraceptive counselling (e.g. brief sexuality-related communication;	423	215 (51)	29 (7)	84 (20)	20 (5)	37 (9)	25 (6)	11 (3)	2 (0)

TYPE OF INTERVENTION: Interventions in Questionnaire Order (Q01-Q86)	Total Respond-ents N	ESSENTIAL in SHSs		HIGHLY SUITABLE in SHSs		SUITABLE in SHSs		UNSUITABLE in SHSs anywhere N (%)	Do not know N (%)
		everywhere N (%)	in certain geographic areas only N (%)	everywhere N (%)	in certain geographic areas only N (%)	everywhere N (%)	in certain geographic areas only N (%)		
counselling on contraception to enable a voluntary, informed choice; referral or provision of contraception if requested post-counselling)[a]									
Q52. Counselling on HIV or other sexually-transmitted infection prevention methods (e.g. brief sexuality-related communication; counselling on correct condom use to enable a voluntary, informed choice; referral or provision of condoms if requested post-counselling)[b]	423	228 (54)	30 (7)	85 (20)	14 (3)	33 (8)	24 (6)	7 (2)	2 (0)

TYPE OF INTERVENTION: Interventions in Questionnaire Order (Q01-Q86)	Total Respond-ents N	ESSENTIAL in SHSs		HIGHLY SUITABLE in SHSs		SUITABLE in SHSs		UNSUITABLE in SHSs anywhere N (%)	Do not know N (%)
		everywhere N (%)	in certain geographic areas only N (%)	everywhere N (%)	in certain geographic areas only N (%)	everywhere N (%)	in certain geographic areas only N (%)		
Q53. Referral and support for voluntary medical male circumcision[a]	423	66 (16)	58 (14)	46 (11)	63 (15)	46 (11)	77 (18)	45 (11)	22 (5)
Q54. Referral and support for HIV prophylaxis[a]	423	125 (30)	70 (17)	64 (15)	53 (13)	41 (10)	45 (11)	14 (3)	11 (3)
Q55. Referral and support for HIV counselling and testing[a]	423	141 (33)	74 (17)	69 (16)	46 (11)	36 (9)	36 (9)	13 (3)	8 (2)
**GENERAL CARE**									
Q56. Provision of first aid, i.e. identification and prioritization of problems, provision of immediate care, and referral for full medical treatment, if required (e.g. acute conditions such as asthma, diabetes, seizures; bleeding or	422	278 (66)	16 (4)	65 (15)	7 (2)	38 (9)	8 (2)	7 (2)	3 (1)

TYPE OF INTERVENTION: Interventions in Questionnaire Order (Q01-Q86)	Total Respond-ents N	ESSENTIAL in SHSs		HIGHLY SUITABLE in SHSs		SUITABLE in SHSs		UNSUITABLE in SHSs anywhere N (%)	Do not know N (%)
		everywhere N (%)	in certain geographic areas only N (%)	everywhere N (%)	in certain geographic areas only N (%)	everywhere N (%)	in certain geographic areas only N (%)		
injury; mental health concerns, including self-harm; life-threatening allergy; poisoning and envenoming; substance abuse)[a]									
Q57. Administration of over-the-counter and prescribed medications[a]	422	133 (32)	26 (6)	83 (20)	26 (6)	46 (11)	35 (8)	56 (13)	17 (4)
Q58. Referral and support for pain control and management[a]	422	152 (36)	23 (5)	95 (23)	20 (5)	73 (17)	25 (6)	22 (5)	12 (3)
Q59. Referral and support for management of non-specific symptoms (e.g. diarrhoea, fever)[a]	422	171 (41)	34 (8)	96 (23)	25 (6)	49 (12)	26 (6)	14 (3)	7 (2)
Q60. Implementation of a health-facility risk management plan linked with	422	188 (45)	28 (7)	83 (20)	20 (5)	52 (12)	16 (4)	16 (4)	19 (5)

TYPE OF INTERVENTION: Interventions in Questionnaire Order (Q01-Q86)	Total Respond-ents N	ESSENTIAL in SHSs		HIGHLY SUITABLE in SHSs		SUITABLE in SHSs		UNSUITABLE in SHSs anywhere N (%)	Do not know N (%)
		everywhere N (%)	in certain geographic areas only N (%)	everywhere N (%)	in certain geographic areas only N (%)	everywhere N (%)	in certain geographic areas only N (%)		
primary, secondary and tertiary care systems (e.g. protocol if school health services should provide essential services during complex emergencies)[a]									
**COMMUNICABLE DISEASES CARE**									
Q61. Referral and support for management of common infections (e.g. ear, eye, skin, throat, urinary tract)[a]	421	210 (50)	25 (6)	85 (20)	11 (3)	52 (12)	19 (5)	15 (4)	4 (1)
Q62. Referral and support for management of less common infectious diseases (e.g. bone infections, cholera, dengue, dysentery, helminths, joint	421	99 (24)	85 (20)	59 (14)	68 (16)	41 (10)	43 (10)	19 (5)	7 (2)

TYPE OF INTERVENTION: Interventions in Questionnaire Order (Q01-Q86)	Total Respond-ents N	ESSENTIAL in SHSs		HIGHLY SUITABLE in SHSs		SUITABLE in SHSs		UNSUITABLE in SHSs anywhere N (%)	Do not know N (%)
		everywhere N (%)	in certain geographic areas only N (%)	everywhere N (%)	in certain geographic areas only N (%)	everywhere N (%)	in certain geographic areas only N (%)		
infections, malaria, meningitis, other neglected tropical diseases, pertussis, pneumonia, rheumatic fever, septicaemia, typhoid fever, tuberculosis, viral encephalitis)[a]									
Q63. Management of infectious disease outbreaks in school, including surveillance, reporting suspected outbreaks to health authorities, and following isolation or quarantine protocols (e.g. cholera; conjunctivitis; dysentery; hand, foot and mouth disease; influenza; meningococcal	421	254 (60)	37 (9)	48 (11)	25 (6)	25 (6)	21 (5)	7 (2)	4 (1)

TYPE OF INTERVENTION: Interventions in Questionnaire Order (Q01-Q86)	Total Respond-ents N	ESSENTIAL in SHSs		HIGHLY SUITABLE in SHSs		SUITABLE in SHSs		UNSUITABLE in SHSs anywhere N (%)	Do not know N (%)
		everywhere N (%)	in certain geographic areas only N (%)	everywhere N (%)	in certain geographic areas only N (%)	everywhere N (%)	in certain geographic areas only N (%)		
disease; rubella; scabies; scarlet fever; tuberculosis; typhoid; varicella)[a]									
**NON-COMMUNICABLE CONDITIONS CARE**									
Q64. Referral and support for chronic care of HIV-infected children[a]	420	127 (30)	96 (23)	47 (11)	61 (15)	31 (7)	36 (9)	16 (4)	6 (1)
Q65. Referral and support for management of anaemia (e.g. iron supplementation)[a]	420	138 (33)	73 (17)	71 (17)	45 (11)	44 (10)	28 (7)	13 (3)	8 (2)
Q66. Referral and support for management of asthma[a]	420	168 (40)	35 (8)	107 (25)	22 (5)	50 (12)	19 (5)	13 (3)	6 (1)
Q67. Referral and support for management of other chronic	420	167 (40)	32 (8)	101 (24)	21 (5)	52 (12)	22 (5)	18 (4)	7 (2)

TYPE OF INTERVENTION: Interventions in Questionnaire Order (Q01-Q86)	Total Respond-ents N	ESSENTIAL in SHSs		HIGHLY SUITABLE in SHSs		SUITABLE in SHSs		UNSUITABLE in SHSs anywhere N (%)	Do not know N (%)
		everywhere N (%)	in certain geographic areas only N (%)	everywhere N (%)	in certain geographic areas only N (%)	everywhere N (%)	in certain geographic areas only N (%)		
conditions (e.g. developmental disabilities/delay, diabetes, heart disease, seizures, sickle cell disease)[a]									
Q68. Referral and support for rehabilitation, habilitation, assistive technology, assistance and support services for injured or disabled individuals (e.g. those who are visually or hearing impaired, who have physical disabilities or motor disorders, or who have sports injuries)[a]	420	186 (44)	25 (6)	88 (21)	23 (5)	53 (13)	22 (5)	16 (4)	7 (2)
**INJURY AND VIOLENCE CARE**									

TYPE OF INTERVENTION: Interventions in Questionnaire Order (Q01-Q86)	Total Respond-ents N	ESSENTIAL in SHSs		HIGHLY SUITABLE in SHSs		SUITABLE in SHSs		UNSUITABLE in SHSs anywhere N (%)	Do not know N (%)
		everywhere N (%)	in certain geographic areas only N (%)	everywhere N (%)	in certain geographic areas only N (%)	everywhere N (%)	in certain geographic areas only N (%)		
Q69. Referral and support for management of common childhood injuries (e.g. head, chest and abdominal injuries; fractures; wounds)[a]	418	241 (58)	12 (3)	82 (20)	5 (1)	52 (12)	7 (2)	14 (3)	5 (1)
Q70. Referral and support for management of burns[a]	418	201 (48)	13 (3)	89 (21)	11 (3)	64 (15)	12 (3)	19 (5)	9 (2)
Q71. Referral and support for management of non-fatal drowning and related complications (e.g. respiratory impairment)[a]	418	169 (40)	32 (8)	78 (19)	19 (5)	61 (15)	18 (4)	28 (7)	13 (3)
Q72. Referral and support for victims of violence (e.g. child abuse and neglect by parents or other caregivers; collective	418	269 (64)	18 (4)	67 (16)	11 (3)	36 (9)	7 (2)	5 (1)	5 (1)

TYPE OF INTERVENTION: Interventions in Questionnaire Order (Q01-Q86)	Total Respond-ents N	ESSENTIAL in SHSs		HIGHLY SUITABLE in SHSs		SUITABLE in SHSs		UNSUITABLE in SHSs anywhere N (%)	Do not know N (%)
		everywhere N (%)	in certain geographic areas only N (%)	everywhere N (%)	in certain geographic areas only N (%)	everywhere N (%)	in certain geographic areas only N (%)		
violence; gender-based or sexual violence; harmful cultural practices; violence among adolescents; and violence by intimate partners)									
**SEXUAL AND REPRODUCTIVE HEALTH CARE**									
Q73. Referral and support for management of pregnancy[a]	418	222 (53)	30 (7)	76 (18)	18 (4)	37 (9)	22 (5)	10 (2)	3 (1)
Q74. Referral and support for management of sexually-transmitted infection[a]	418	217 (52)	27 (6)	84 (20)	14 (3)	40 (10)	21 (5)	12 (3)	3 (1)
**MENTAL HEALTH CARE**									

TYPE OF INTERVENTION: Interventions in Questionnaire Order (Q01-Q86)	Total Respond-ents N	ESSENTIAL in SHSs		HIGHLY SUITABLE in SHSs		SUITABLE in SHSs		UNSUITABLE in SHSs anywhere N (%)	Do not know N (%)
		everywhere N (%)	in certain geographic areas only N (%)	everywhere N (%)	in certain geographic areas only N (%)	everywhere N (%)	in certain geographic areas only N (%)		
Q75. Provide short-term counselling or crisis intervention focused on mental health or situational concerns (e.g. grief, difficult transitions)[a]	418	221 (53)	16 (4)	100 (24)	10 (2)	45 (11)	15 (4)	8 (2)	3 (1)
Q76. Referral and support for child carers (e.g. students who provide unpaid support to a parent who could not manage without this help)	418	148 (35)	22 (5)	95 (23)	33 (8)	69 (17)	28 (7)	15 (4)	8 (2)
Q77. Referral and support for management of common behavioural disorders in children (e.g. Attention Deficit Hyperactivity Disorder)[a]	418	226 (54)	13 (3)	94 (22)	13 (3)	47 (11)	12 (3)	9 (2)	4 (1)

TYPE OF INTERVENTION: Interventions in Questionnaire Order (Q01-Q86)	Total Respond-ents N	ESSENTIAL in SHSs		HIGHLY SUITABLE in SHSs		SUITABLE in SHSs		UNSUITABLE in SHSs anywhere N (%)	Do not know N (%)
		everywhere N (%)	in certain geographic areas only N (%)	everywhere N (%)	in certain geographic areas only N (%)	everywhere N (%)	in certain geographic areas only N (%)		
Q78. Referral and support for management of emotional, anxiety and depressive disorders[a]	418	239 (57)	11 (3)	92 (22)	9 (2)	47 (11)	12 (3)	3 (1)	5 (1)
Q79. Referral and support for management of eating disorders (e.g. anorexia, bulimia)[a]	418	184 (44)	24 (6)	96 (23)	19 (5)	62 (15)	19 (5)	6 (1)	8 (2)
Q80. Referral and support for management of stress[a]	418	202 (48)	13 (3)	117 (28)	6 (1)	59 (14)	11 (3)	7 (2)	3 (1)
Q81. Referral and support for management of suicide risk/self-harm	418	259 (62)	19 (5)	77 (18)	8 (2)	36 (9)	12 (3)	2 (0)	5 (1)
Q82. Referral and support for management of somatoform disorders (i.e. physical symptoms that suggest	418	138 (33)	20 (5)	115 (28)	8 (2)	72 (17)	21 (5)	30 (7)	14 (3)

TYPE OF INTERVENTION: Interventions in Questionnaire Order (Q01-Q86)	Total Respond-ents N	ESSENTIAL in SHSs		HIGHLY SUITABLE in SHSs		SUITABLE in SHSs		UNSUITABLE in SHSs anywhere N (%)	Do not know N (%)
		everywhere N (%)	in certain geographic areas only N (%)	everywhere N (%)	in certain geographic areas only N (%)	everywhere N (%)	in certain geographic areas only N (%)		
illness or injury, but which cannot be explained fully by a general medical condition or by the direct effect of a substance)[a]									
Q83. Referral and support for management of psychotic disorders[a]	418	190 (45)	20 (5)	80 (19)	8 (2)	65 (16)	16 (4)	27 (6)	12 (3)
**SUBSTANCE USE CARE**									
Q84. Referral and support for management of harmful use of a substance (e.g. alcohol, illicit drugs)[a]	418	224 (54)	18 (4)	99 (24)	6 (1)	43 (10)	16 (4)	8 (2)	4 (1)
Q85. Referral and support for management of dependence on a substance (e.g. alcohol, illicit drugs)[a]	418	211 (50)	17 (4)	100 (24)	12 (3)	44 (11)	16 (4)	11 (3)	7 (2)

TYPE OF INTERVENTION: Interventions in Questionnaire Order (Q01-Q86)	Total Respond-ents N	ESSENTIAL in SHSs		HIGHLY SUITABLE in SHSs		SUITABLE in SHSs		UNSUITABLE in SHSs anywhere N (%)	Do not know N (%)
		everywhere N (%)	in certain geographic areas only N (%)	everywhere N (%)	in certain geographic areas only N (%)	everywhere N (%)	in certain geographic areas only N (%)		
Q86. Referral and support for management of substance withdrawal[a]	418	180 (43)	22 (5)	93 (22)	11 (3)	58 (14)	18 (4)	25 (6)	11 (3)

Key: HEADSSS=home, education/employment, eating, activity, drugs, sexuality, safety, and suicidal thinking / depression; HIV=Human Immunodeficiency Virus; Q##=Questionnaire number/order

[a] Post-coded as clinical interventions that must be delivered by or supervised by a health worker.

[b] Post-coded as clinical interventions that could be delivered by a health worker but might be delegated to a teacher with health worker support or supervision.
