# Supplementary material for: Results of a Global Survey of Experts to Categorize the Suitability of Interventions for Inclusion in School Health Services
Source: J Adolesc Health. 2021 Dec;69(6):948–56. doi: 10.1016/j.jadohealth.2021.05.008 (PMC8631416; doi:10.1016/j.jadohealth.2021.05.008)
Supplement: Appendix 2 [file mmc2.pdf]

Results of a Global Survey of Experts to Categorize the Suitability of Interventions for Inclusion in School Health Services

Appendix 2

Examples of clinical intervention suggested by 1-3 respondents, by type of intervention.[a]

ASSESSMENT
Systematic assessments at the beginning of the school year and quarterly for common illnesses
**SCREENING**
End ineffective screenings
**MASS DRUG ADMINISTRATION AND IMMUNIZATION**
HPV vaccination for men
**HEALTH COUNSELING TO PROMOTE WELL-BEING**
Use of expressive arts to assist learners with general skills such as assertiveness, handling emotions and conflict resolution to prevent violent behaviour, particularly essential in geographical areas where violence rates are higher[b]
**GENERAL CARE**
Management of heat-related symptoms in places where temperatures are high in classrooms due to an unsuitable built environment[b]
**NONCOMMUNICABLE CONDITIONS CARE**
Support and management of allergies and anaphylaxis

INJURY AND VIOLENCE CARE
Referral and support for female genital mutilation (detected, suspected, or at-risk)[c]
**SEXUAL AND REPRODUCTIVE HEALTH CARE**
Sexual and reproductive health care for the lesbian, gay, bisexual, transgender, and intersex populations
**MENTAL HEALTH CARE**
Family emotional health management (psychological counseling for children when parents are divorced)
**SUBSTANCE USE CARE**
Obtaining updates on modern recreational drugs

[a] 119 interventions were suggested by 1-3 respondents. All interventions shown here were suggested for inclusion in SHSs everywhere, unless otherwise noted.

[b] Suggested for SHSs in certain geographic areas only.

[c] Suggested both for SHSs everywhere and for SHSs in certain geographic areas only.
